# An Evaluation of Language Preference between Spanish and English with Picture Exchange Users

**DOI:** 10.1007/s40617-025-01141-1

**Published:** 2026-01-05

**Authors:** Alexandra N. Mercado Baez, Tyler-Curtis C. Elliott, Ana Grullon, Kevin M. Ayres

**Affiliations:** Rivers Crossing, 850 College Station Road Bldg. 2, Athens, GA 30605 USA

**Keywords:** Picture exchange communication system, Language preference assessment, Concurrent schedule arrangement

## Abstract

Picture-based communication systems can effectively convey meaning to a listener regardless of the listener's language fluency. In other words, picture-based communication systems can serve as a means of cross-lingual communication. The current study employed a concurrent schedule assessment to measure the allocation of responses between two languages of four pre-kindergarten students with autism. We reversed contingencies (i.e., switched the language with which we responded) to ensure the allocation was due to the researcher's language use rather than some other variable (i.e., preference for the left side). Results showed three response patterns: two students consistently allocated to Spanish (i.e., had a preference), one student consistently chose pictures from the left side regardless of the language (suggesting either a side bias or no language preference), and one student began allocating to one language but did not maintain this allocation following several contingency reversals. These results suggest that users of picture-based augmentative and alternative communication systems may prefer the language used by their communication partner, and that a concurrent schedule arrangement can help identify the student's language preference. We discuss limitations and future directions for assessing language preferences.

Board-certified behavior analysts (BCBAs) have ethical requirements related to cultural responsiveness and diversity, which include considering their clients’ needs regarding language and language preference (Behavior Analyst Certification Board [BACB], 2020, 1.07, 2.14). Jimenez-Gomez and Beaulieu (2022) emphasized that understanding how language and cultural factors intersect is particularly crucial when working with bilingual families, because cultural responsiveness is essential for delivering high-quality behavior-analytic services when providers and recipients have different linguistic or cultural backgrounds**.** Moore and Pérez-Méndez (2006) highlighted that many parents and professionals who work in early intervention have assumed that individuals with speech or language delays cannot learn two languages or that exposure to two languages will delay language development. However, Drysdale et al. (2015) reported in a systematic review that seven out of eight studies failed to show that raising children with autism spectrum disorder (ASD) in a multilingual environment impeded language development. Furthermore, Lim et al. (2018) noted that the emerging literature does not support this statement and that there has not been any empirical support for this statement. Individuals with developmental disabilities living in locations where their first language is not spoken may be disadvantaged if their schooling does not account for their need to communicate in their current locale while maintaining their heritage language. Learning only one language may restrict their ability to connect with a significant part of their verbal community, such as their church and extended family members (IRIS Center, 2015).

Current research suggests that for some learners with developmental disabilities, the language spoken during instruction can affect functional analysis outcomes (Neely et al., 2020; Rispoli et al., 2011), success with functional communication training (Neely et al., 2020; Padilla Dalmau et al., 2011), engagement in play (Lim & Charlop, 2018), accuracy during instruction (Lang et al., 2011), and skill acquisition during bilingual instruction (León & Rosales, 2018). These findings underscore the importance of considering a client's language background and suggest that identifying a learner’s language preference may be a crucial step in research and clinical practice.

Although one straightforward way to assess language preference is to ask the individual their preference, researchers have used behavioral methodologies to obtain more objective measures of these preferences in polyglot clients. One approach involves the use of concurrent‐chain arrangements to evaluate language choice under controlled conditions (Aguilar et al., 2016). In their study, Aguilar et al. (2016) employed a choice arrangement to determine participants' language preferences for instruction, in particular between English, Spanish, and a no-language-control condition, using a concurrent-operant design. The researchers provided participants with the option to select English, Spanish, or a no-language control condition using communication devices. The results indicated that concurrent operant arrangements were adequate for assessing language preference during instruction for learners with autism in the study.

In addition, Clay et al. (2020) conducted a concurrent operant reinforcer assessment to evaluate the effectiveness of praise in different languages during instruction delivery. In this assessment, participants completed identical tasks, each linked to a different type of praise: no praise, praise in English, and praise in Spanish. The results showed that participants demonstrated little to no preference for one language over the other. Last, Kunze et al. (2019) explored preferences for language output on a speech-generating device (SGD) and the preferred language of the interventionist for a client with Down syndrome. Kunze et al. (2019) involved three SGDs placed on the table, each representing one of the three language output options: English, Spanish, or a control condition. After the student made a request, the researcher removed the devices until the student finished consuming their chosen edible item. The researcher then reintroduced the devices and continued the process until the session concluded. Throughout the interaction, the researchers communicated with the participant in the language the participant chose.

The Picture Exchange Communication System (PECS) is an alternative and augmentative communication (AAC) method used to teach individuals to exchange pictures as a verbal response with a communication partner (Frost & Bondy, 2002). Picture exchange, a low-tech strategy, offers benefits for dual language learners because it utilizes pictures that can be universally interpreted and reinforced by listeners of any language. However, just because picture exchange can be cross-lingual, it does not mean that language (i.e., Spanish, English) has no impact on the individual's need for a communication partner. We were unable to identify a published study evaluating user preference for PECS in relation to the language of their communication partner. However, Kunze et al. (2019) evaluated language output and preference for a speech-generating device, as well as the interventionist's language usage. The results suggest that participants preferred the Spanish language in the device output. We cannot assume, however, that the findings of Kunze et al. (2019) will apply to picture exchange users who do not have direct speech output, other than that of their communication partner. SGDs are unique because, following a selection, the device immediately generates output. The AAC user then has to wait for the communication partner to respond. If the user's device outputs in Spanish and the communication partner is a monolingual English speaker, this may cause a communication breakdown. Picture exchange is unique in that a dual language learner can exchange a picture, and a listener can understand the request in any language (due to a communication partner serving as the "voice output"). Because of this, we aimed to evaluate the effects of communication partner language usage on picture exchange allocation during concurrent schedule arrangements with the following research questions: (1) What are the effects of the language used by the communication partner on picture exchange allocation in a concurrent schedule arrangement? and (2) When the contingencies are reversed (i.e., the language type used for each schedule changes by the communication partner) will the participant continue to demonstrate a preference for one language over the other (i.e., can this arrangement detect language preference)?

## Method

### Participants and Setting

Participants included four pre-kindergarten students in a classroom located in a Southeastern public school, which the local university operated as a practicum site for training future behavior analysts. All sessions took place in the classroom work area, which consisted of two rectangular tables and four child-sized chairs. To participate, students had to be dual language learners, have Spanish as their primary language spoken at home, and have mastered PECS Phases 1-3B (Frost & Bondy, 2002). The first three authors were teachers in the students' classrooms and ran all sessions. The authors typically communicated with the participants in English at school. The first and third authors were Latina women who spoke Spanish as their native language. The second author had a bachelor's degree in Spanish and spoke Spanish as a second language. We conducted sessions 1 to 4 days a week (depending on student availability) with no more than four sessions per day.

Jose, Hugo, Rafael, and Santana were 4-year-old Latino males with school eligibility for autism spectrum disorder (ASD). Jose, Hugo, and Santana primarily communicated through picture exchange. Rafael primarily communicated using a combination of two- and three-sentence words, as well as picture exchange. Hugo scored a 47 on the cognitive domain of the Developmental Profile-Third Edition (DP-3; Alpern, 2007) evaluation and a zero on the Early Echoic Skills Assessment (EESA; Esch, 2008). Rafael scored a 75 on the cognitive domain of the DP-4 and a 94 on the EESA. Jose scored less than 50 on the cognitive domain of the DP-3 and a zero on the EESA. Santana did not have a DP-3 score; he scored a 64 on the EESA.

### Materials

Materials included a laminated piece of paper (referred to as the communication board), which was separated down the middle into yellow and blue sides with Velcro to hold the picture cards in place (see Fig. 1). The size, shape, and Velcro positions were identical between the left and right sides. Each side of the board was a different color to promote discrimination. The number of preferred items varied for each participant based on the availability and accessibility of deliverable reinforcers in the classroom. To keep the array on the board a manageable size, we limited it to five pictures. We created two sets: one with the item's name in English and the other with the item's name in Spanish. Apart from the language used on the item's label, the pictures were identical.

**Fig. 1:**
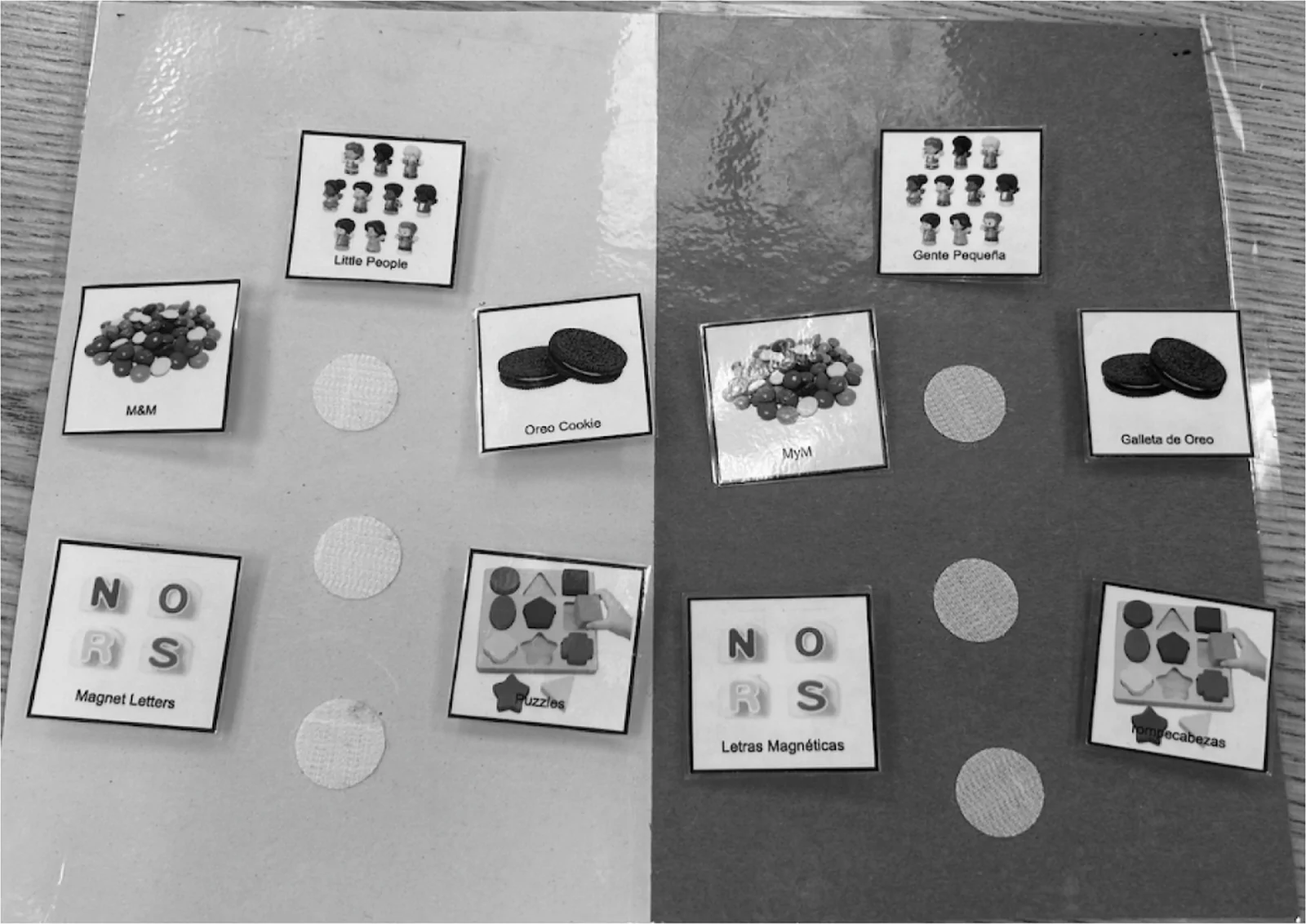
Example of the concurrent schedule board arrangement

### Response Definitions and Measurement

Each session consisted of 10 trials; a trial ended once the student engaged with the leisure item for 30 s or consumed the edible. If the student grabbed a picture and exchanged it with the communication partner but did not engage with the item or consume the edible, we assumed this was a discrimination error. Thus, we did not include the trial in our count. The student had another opportunity to exchange the picture of another item or edible. We calculated the percentage of response allocation to each language by dividing the number of trials where students chose the left or right side (each representing either English or Spanish) by 10 and then multiplying by 100%.

### Reliability

A secondary observer collected data for both participants across all conditions. The training for the secondary observer included scoring in-vivo sessions until they achieved at least 90% agreement with the primary observer. We calculated interobserver agreement (IOA) by using point-by-point agreement (Ledford & Gast, 2024). At the end of the session, we counted the instances in which both data collectors agreed on the side the student selected, then divided these instances by the total number of trials and multiplied by 100 (Ledford & Gast, 2024). The secondary observer coded 31.25% of sessions for Hugo, 37.03% of sessions for Rafael, 33.33% of sessions for Jose, and 27.78% of sessions for Santana. All sessions for all participants had 100% agreement.

### Experimental Design

We implemented a reversal design with an embedded concurrent schedule using a communication board with two clearly discriminable responses (i.e., selections from the left or right), each signaled by a discriminative stimulus (i.e., yellow or blue), each corresponding to a specific contingency (i.e., responses in Spanish or English). Because allocation to one side of the board (e.g., left) could be due to a factor other than the language type consequence (e.g., a preference for the color itself or a left-side bias), we reversed the contingencies associated with each side of the board (Gast et al., 2019). That is, if in the first phase, selecting from the left side of the board resulted in an item and a response in Spanish, then in the second phase, selecting from the left side of the board would result in the item and a response in English. We initiated this reversal when the student allocated 60% or more selections to one side of the board for three consecutive sessions, with stability in the data (i.e., no trend), or, if the student did not meet this criterion, after 10 sessions. To control for cyclical variability (e.g., having three sessions in each phase of the design), when the student met the three-session criteria, we conducted an additional session. Last, we counterbalanced the color assignment across students, specifying which color represented each side (which remained constant throughout the study), and which language consequence initiated on each side (which switched during the reversal).

### Procedure

#### General Procedure

During all sessions, we placed the pictures of the students' preferred items on the communication board arrangement. For all sessions, when the student exchanged the picture, the student received the requested item, and the therapist named the item in the corresponding language. In both the English and Spanish sessions, we presented the contingencies in English, and the spoken prompt remained consistent across languages, as the student heard English throughout the entire school day. While the student engaged with the item or edible, we interacted with the student in the language matching their selection. For example, if the students selected a picture of a leisure item on the Spanish side of the board, we named the item in Spanish (e.g., for train, we said "tren"), interacted with the student in Spanish by narrating the student interaction with the item in Spanish (e.g., "el tren es rojo"; "el tren va muy rápido"), and praised the student for asking for the item in that language (e.g., "buen trabajo"; "gracias por preguntar”). If they selected the picture of a leisure item on the English side of the board, we named the item in English (e.g., "train"), praised the student for asking for the item (e.g., "good job"; "thank you for asking"), interacted with the student in English by narrating the student interaction in English (e.g., "the train is red"; "the train goes so fast"), and started a timer for the student to engage with the item.

In addition, if the student selected the picture on the English side of the board, we named the edible in English (e.g., cookies) and interacted with the student in that language (e.g., "yummy, you are eating a cookie"; "cookie looks delicious"). We allowed the student sufficient time to eat the edibles (approximately 5 s). Once the student finished eating the edible, we placed the card back in its original location. In addition, during all sessions, all leisure items and edibles were placed on the table, ensuring they were always visible to the student.

#### Exposure to the Contingencies

At the beginning of the study, and then again, each time we reversed the contingencies (switched the language we responded with), we exposed the students to the contingencies for each side of the board. We randomly selected the starting language (e.g., English or Spanish). We placed one set of pictures on their predetermined side of the board, stating the contingency, "When you hand me the card from the [color] side, I will talk to you in [language]." Thus, during this condition, the participants had the communication board in front of them. However, only one side included pictures, forcing the student to consistently select the language (i.e., side) for 10 trials. After 10 trials, we did the same with the other language on the opposite side of the board.

#### Concurrent Schedule Arrangement

We placed each set of pictures (English and Spanish) on its predetermined side of the board. We put the communication board in front of the student and stated the contingency: "When you hand me the card from the [color] side, I will talk to you in Spanish. When you hand me the card from the [other color] side, I will talk to you in English."

### Procedural Fidelity

To measure researcher fidelity during sessions, we used two unique checklists, one that listed the critical steps for exposing participants to the contingencies condition and one that listed the essential steps for the concurrent schedule arrangement. The first checklist included five steps. First, the picture cards were on the correct side of the arrangement, depending on the target language. Second, the instructor provided the student with the requested item. Third, the instructor accepted the picture and named the item in the targeted language. Fourth, the instructor interacted with the students in the targeted language. Last, if the student selected a leisure item, the instructor started a 30-s timer.

The second checklist included four steps for the concurrent schedule arrangement. First, the implementer placed all the picture cards on the correct side of the arrangement (e.g., Spanish on the right side, English on the left side, or vice versa). Second, the instructor provided the student with the requested item. Third, the instructor accepted the picture and named the item in the target language. Last, while the student engaged with the item or edible, the instructor interacted with the student in the target language. A secondary observer used these checklists to collect procedural fidelity data for at least 31.25% of sessions in each condition.

For Hugo, the secondary observer collected data for 37.5% of the contingency sessions and 31.25% of the concurrent schedule preference assessment sessions and obtained an average of 98% (range: 90%–100%) of steps followed correctly in both conditions. For Rafael, the secondary observer collected data during 25% of the exposure to the contingency sessions and 37% of the concurrent schedule preference assessment, with an average of 100% of steps followed correctly in both conditions. With Jose, the secondary observer collected data for 37.5% of the contingency sessions and 33.33% of the concurrent schedule preference assessment sessions, with an average of 100% of steps followed correctly in both conditions. With Santana, the secondary observer collected data in 75% of the contingency sessions and 27.78% of the concurrent preference assessment sessions, achieving an average accuracy of 100% in both conditions.

## Results

Figure 2 illustrates the percentage of the language allocation for Hugo. In the first condition, Hugo allocated an average of 93.33% (range: 80%–100%) of the trials to the right side (Spanish) and 6.67% (range: 0%–20%) to the left side (English). After the first reversal of the contingencies, he allocated an average of 83.33% (range: 60%–100%) of trials to the left side (Spanish) and an average of 16.67% (range: 0%–40%) to the right side (English). After the second reversal, Hugo allocated an average of 75% (range: 10%–100%) of the trials to the right side (Spanish) and allocated 25% (range: 0%–90%) to the left side (English). After the third reversal, he allocated an average of 72.5% (range: 10%–100%) of the trials to the left side (Spanish) and 27.5% (range: 0%–90%) to the right side (English). Overall, Hugo allocated 81.04% (range: 10%–100%) of all sessions to Spanish and 18.96% (range: 0%–90%) to English.

**Fig. 2:**
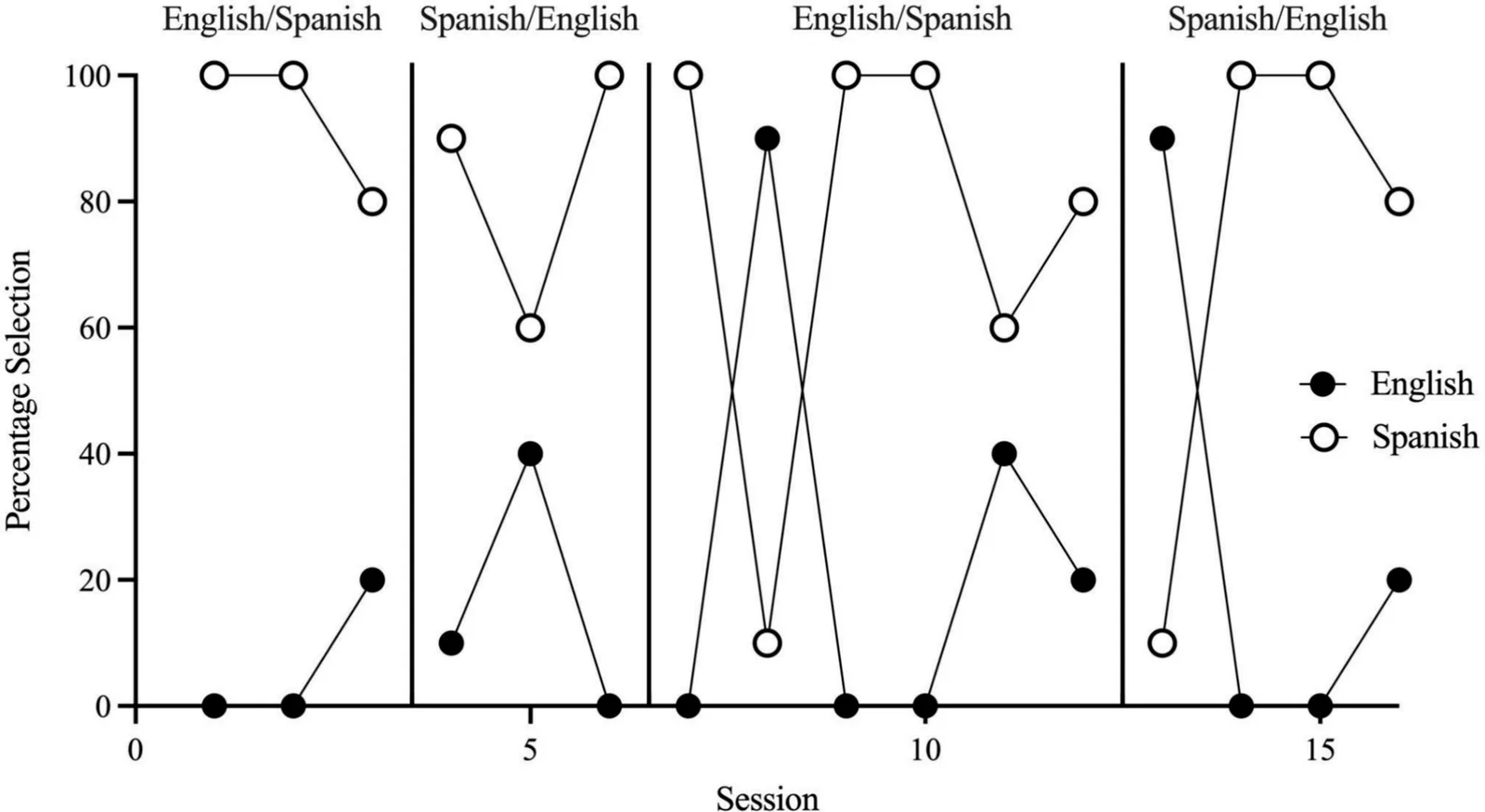
Hugo's Language Preference Assessment Results

Figure 3 displays the percentage of the language allocation for Rafael. In the first condition, Rafael allocated an average of 53.33% (range: 33.33%–70%) of trials to the right side (English) and 46.66% (range: 30%–66.66%) to the left side (Spanish). After the first reversal, Rafael allocated an average of 80% (range: 70%–100% to the left side (English) and 20% (range: 0%–40%) to the right side (Spanish). After consistent data and low levels of variability, we implemented the second reversal. In this condition, Rafael allocated an average of 70% (range: 50%–90%) of trials to the right side (English) and allocated an average of 30% (range: 10%–50%) to the left side (Spanish). After the third reversal, Rafael allocated an average of 63.33% (range: 20%–90%) to the left side (English) and 36.67% (range: 10%–80%) to the right side (Spanish). After the last reversal, Rafael allocated an average of 90% (range: 80%–100%) to the right side and allocated 10% (range: 0%–10%) to the left side (Spanish). Overall, Rafael allocated an average of 66.05% (range: 20%–100%) of trials to English and allocated 33.95% (range: 0%–80%) to Spanish.

**Fig. 3:**
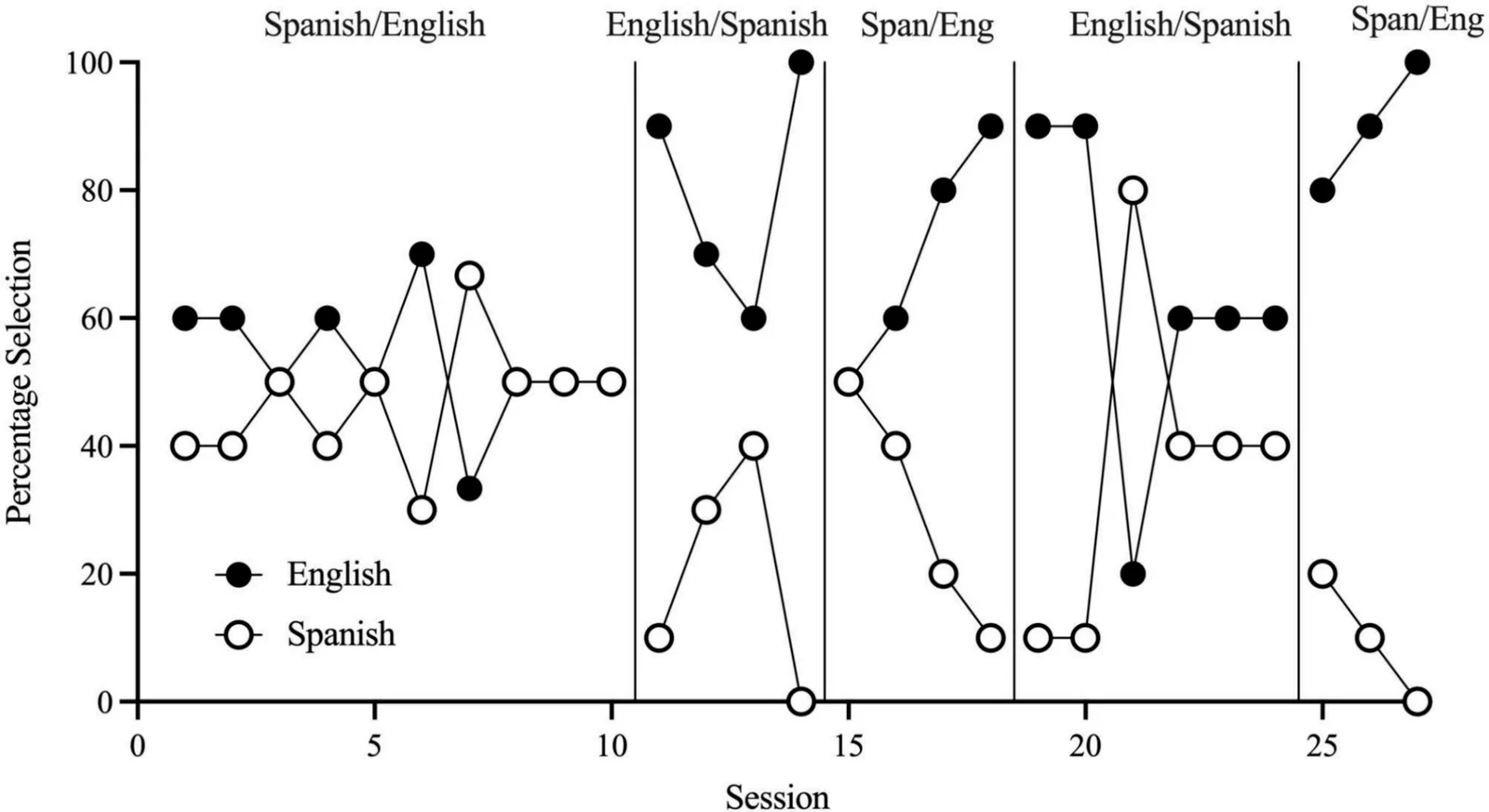
Rafael's Language Preference Assessment Results

Figure 4 depicts the percentage of the language allocation for Jose. In the first condition, Jose allocated an average of 78% (range: 40%–90%) of trials to the right side (Spanish) and 22% (range: 10%–60%) to the left (English). After the first reversal, Jose allocated an average of 77.5% (range: 60%–90%) to the right side (English) and 22.5% (range: 10%–40%) to the left side (Spanish). In this condition, Jose allocated an average of 80% (range: 70%–90%) of trials to the right side (Spanish) and allocated 20% (range: 10%–30%) to the left side. During the third reversal, Jose allocated an average of 83.33% (range: 80%–90%) to the right side (English) and 16.67% (range: 10%–20%) to the left side (Spanish). Overall, Jose allocated an average of 50.71% (range: 10%–90%) of trials to English and 49.29% (range: 10%–90%) to Spanish.

**Fig. 4:**
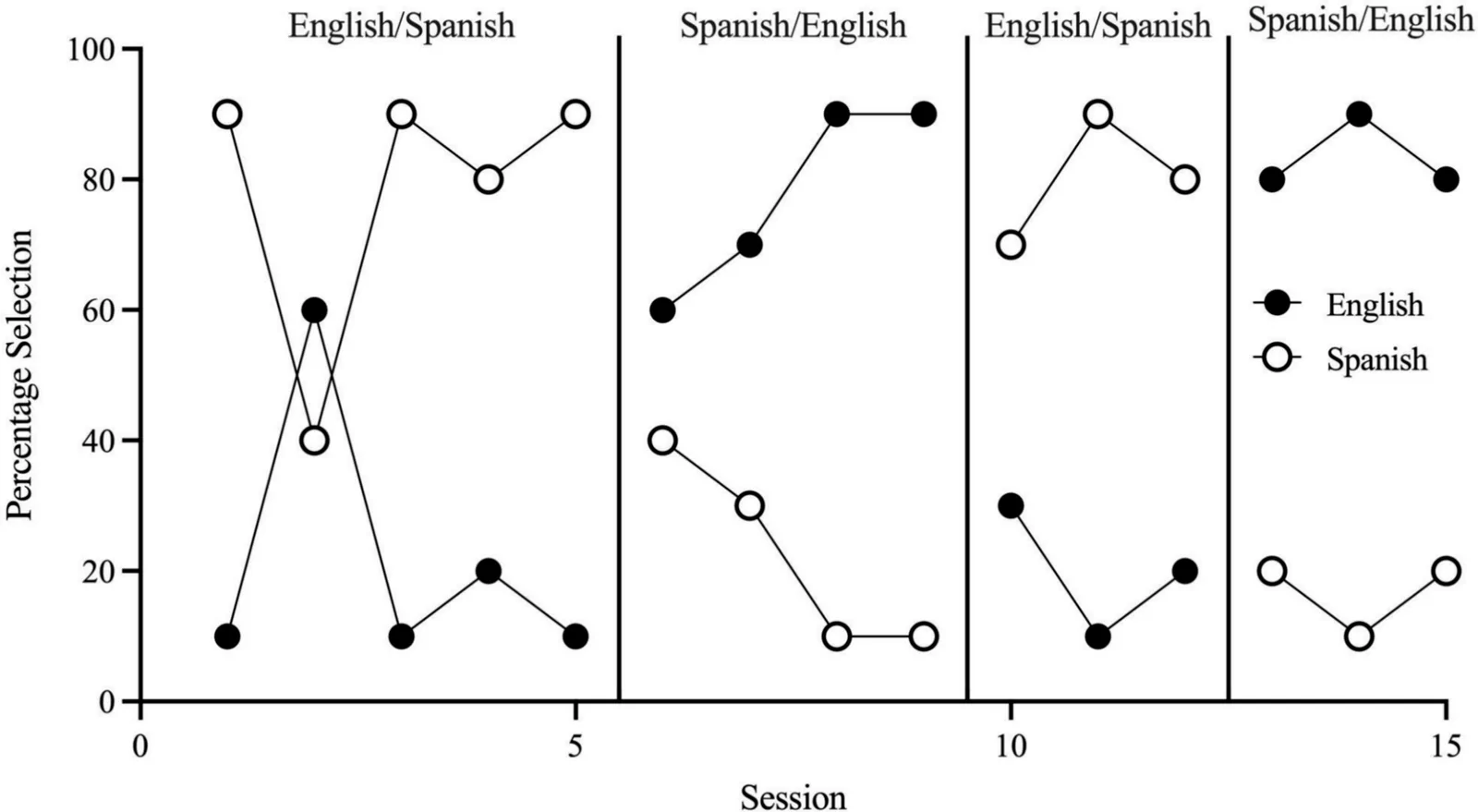
Jose's Language Preference Assessment Results

Figure 5 shows the percentage of the language allocation for Santana. In the first condition, Santana allocated an average of 90% (range: 80%–100%) to the left side (Spanish) and allocated 10% (range: 0%–20%) to the right side (English). After the first reversal, Santana allocated an average of 60% (range: 0%–100%) of trials to the right side (Spanish) and 40% (range: 0%–100%) to the left side (English). After the second reversal, Santana allocated an average of 100% of trials to the left side (Spanish) and 0% to the right side (English). After the third reversal, Santana allocated an average of 70% (range: 30%–100%) of the sessions to the left side (English), and allocated an average of 30% (range: 0%–70%) of the sessions to the right side (Spanish). Overall, Santana allocated an average of 63.89% (range: 0%–100%) of all sessions to Spanish and an average of 36.11% (range: 0%–100%) to English. Figure 6 shows a summary of the average percentage of language selection for each participant.

**Fig. 5:**
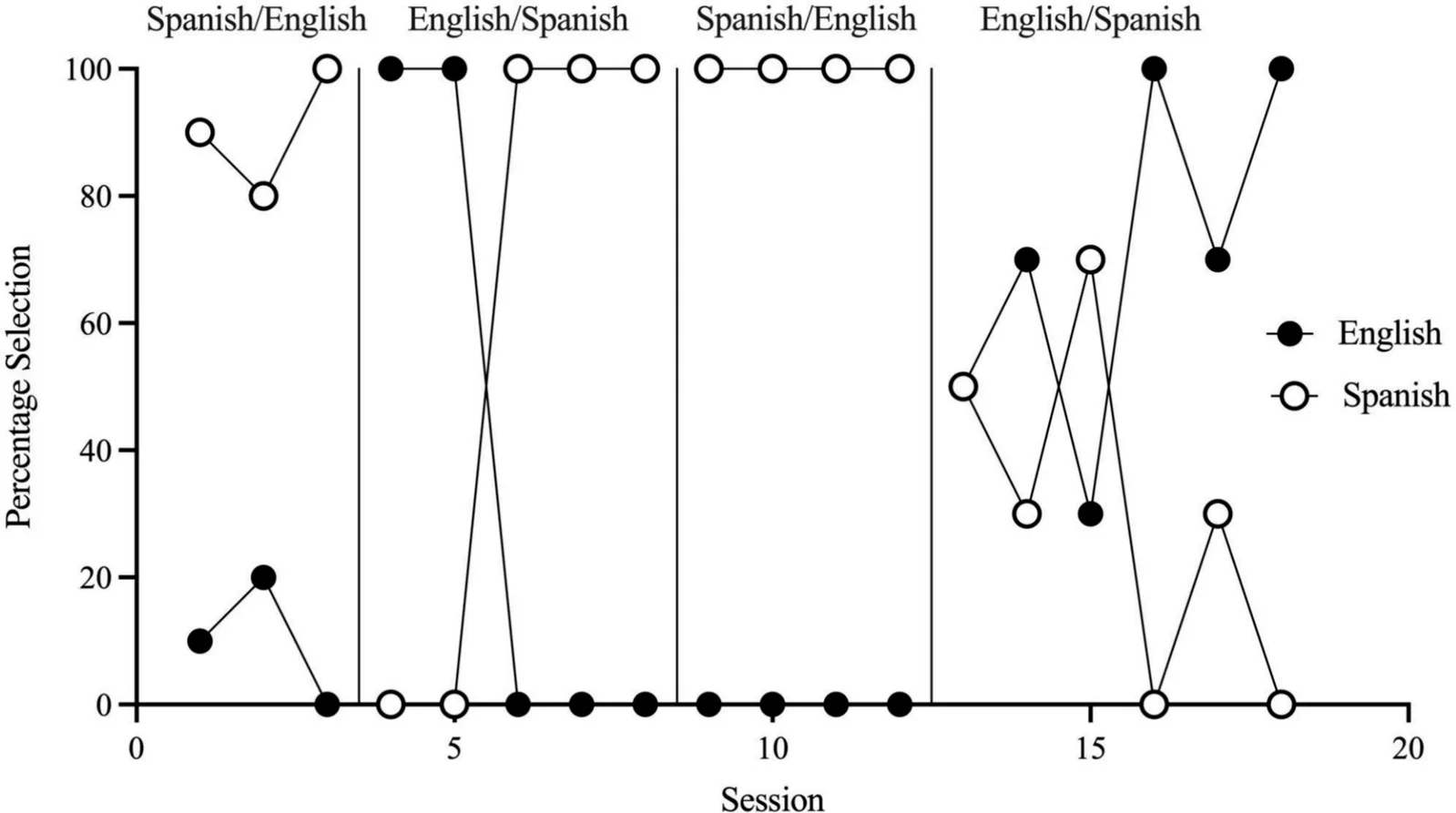
Santana's Language Preference Assessment Results

**Fig. 6:**
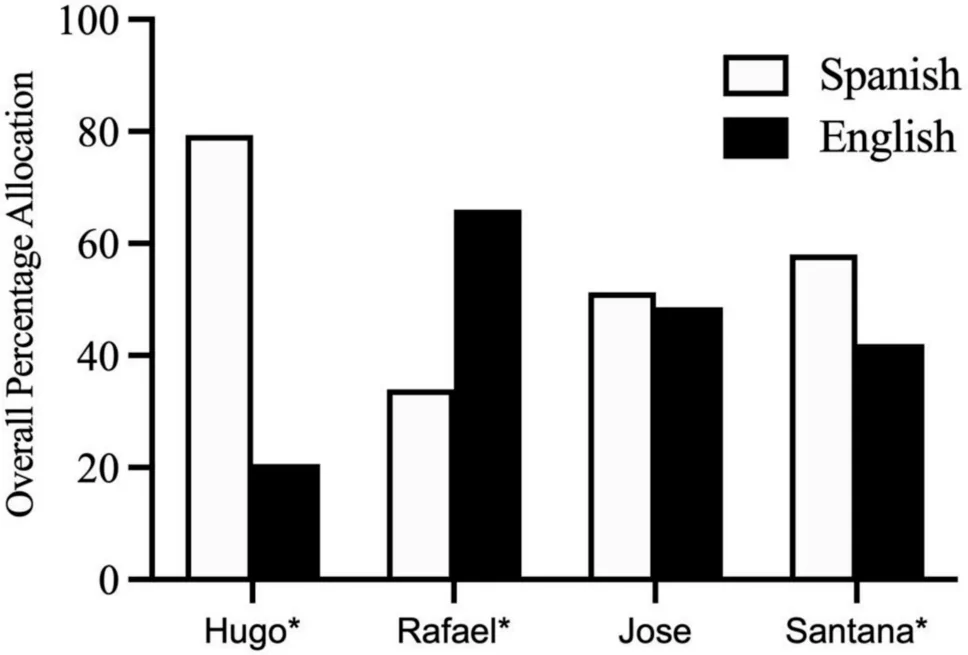
Average Percentage Selection for Each Language

## Discussion

Researchers within the field of behavior analysis have evaluated the effects of exposure to multiple languages on individuals with developmental disabilities (Lang et al., 2011; Lim & Charlop, 2018; Neely et al., 2020; Padilla Dalmau et al., 2011; Rispoli et al., 2011). An emerging body of research has also examined language *preferences* among individuals raised in multilingual households (Aguilar et al., 2016). Kunze et al. (2019) used a picture-based speech-generating device (SGD), and the participants showed a preference for the home language voice output. Picture exchange does not contain a voice output; instead, the communication partner provides the "voice output" for the learner by reading the exchanged card and then responding appropriately. Given this specific difference, the findings of Kunze et al. (2019) may not generalize to PECS users (with no speech output). Because of this, we aimed to evaluate the effects of communication partner language usage on picture exchange allocation during concurrent schedule arrangements. In this arrangement, we presented two languages (English and Spanish) simultaneously, and participants chose the language with which they wanted to interact. Our goal was to use this arrangement to detect language preferences.

We found three different response patterns. The first, as seen by Hugo and Rafael, is that the various language contingencies resulted in consistent allocation towards one specific language (Spanish for Hugo and English for Rafael) in the concurrent schedule arrangement. This response pattern persisted when the implementers reversed the contingencies. That is, the student switched the side of the communication board from which they selected pictures when the side of the language response changed. The reversal provided verification and replication that these students preferred one language over the other. Rafael did not begin allocating to one side (language) until the second exposure to the contingencies, which suggests that some students may require more exposure to the contingencies before implementing the concurrent schedule arrangement.

Jose demonstrated the second response pattern. He consistently chose the left side of the concurrent schedule arrangement, regardless of the language assigned to that side, suggesting that there was no language preference or that Jose may have been subject to some form of side bias. Thus, if a client exhibits a side bias, this current assessment method may yield inconclusive results, potentially leading to false assumptions about a student's language preference. This pattern also highlights the need for replication to ensure that the variable in question (language preference) was due to the therapist's behavior (i.e., the language used) rather than uncontrolled variables.

Santana's responses reflect a third unique pattern. He initially allocated to one language (Spanish). He continued to allocate to that language following the first reversal, but we could not replicate this effect when we reversed the contingency one last time. We are unsure why we did not observe a third demonstration of impact for this participant. It is possible that repeated exposure to one language could have abolished the value of that language (i.e., satiation). Thus, language preferences may not be static. This explanation has merit, given that other researchers have demonstrated that preferences change over time (Melanson et al., 2023). Like other preference assessments, practitioners should periodically conduct language preference assessments to ensure ongoing alignment with participants' preferences.

These results suggest that language preference may be idiosyncratic due to each learner's unique history and experiences with different languages. In other words, an individual's preferred language may depend on their comfort, exposure, and reinforcement history in that language. Likewise, the language instruction should also be individualized, as learners may respond differently depending on their linguistic background and learning history (León & Rosales, 2018).

Behavior analysts conducting studies on language acquisition or providing clinical services to families and individuals with developmental disabilities should consider the individuals' and their families' cultural and linguistic backgrounds. This can play a crucial role in analyzing the behavior of these individuals and families (Brodhead et al., 2014). BCBAs are bound by professional ethics to be culturally responsive and consider their clients' language and cultural needs (BACB, 2020, 1.07). This study extends the findings of Kunze et al. (2019) by using picture-exchange cards to evaluate language preference when the 'voice output' is from someone other than the participant (e.g., the interventionist).

These results demonstrate the utility of low-tech AAC systems in evaluating language preference. The detection of language preference can help determine the course of treatment and should inform the decision-making process for both practitioners and researchers (Neely et al., 2020). Ringdahl et al. (2016) provide another published example of how aspects of communication can affect the individual's preference. In particular, the researchers taught 18 participants functional communication responses using multiple communication modalities (e.g., signs, microswitch, and picture cards). When the researchers evaluated participants' preferences for these modalities using a concurrent operant arrangement, all students eventually allocated to one AAC modality, highlighting participant preferences. Thus, our study contributes to the existing literature, which demonstrates that we can identify the AAC communication preferences (e.g., the language used by communication partners; the modality of the AAC device) of people with developmental disabilities using concurrent operants.

In addition, these findings have significant implications for social validity. In general, when participants have significant language or cognitive impairments, Common and Lane (2017) suggest that using the results of preference assessments to inform treatment is essential for increasing social validity. Thus, in our arrangement, when participants consistently selected one language over the other, this response pattern suggests that the chosen language was more socially valid. With this information, behavior analysts can also set up teaching arrangements that are more socially valid for their clients. For example, the behavior analysts could begin teaching the use of the autoclitic "I want" using PECS Phase 4, but use Spanish when teaching. Then the behavior analyst could assess the generalization of the skill when the communication partner uses English.

### Limitations and Future Research

We encountered several limitations that temper our conclusions. First, we did not conduct either formal or informal interviews with parents before commencing the study. Because the first author spoke Spanish, she communicated with the parents in Spanish during teacher–parent interactions (e.g., check-in, drop-off, pick-ups, parent–teacher conferences). In addition, the second author typically spoke English to the students and their parents daily, but only spoke Spanish to the students during the study. Furthermore, parents indicated on the intake forms that they spoke Spanish at home. However, we did not gather additional information about the frequency with which they spoke their native language or the extent to which the student heard Spanish at home.

Future research should include formal interviews to gain a deeper understanding of participants' language exposure in their home environment. In addition, we suggest that future researchers evaluate the effects of using a preferred language on skill acquisition and behavior reduction programs, as well as the impact of language preference on assessment outcomes (e.g., functional analyses). Last, we suggest that future researchers could empirically evaluate if teaching in alignment with students' preferences leads to improved communication outcomes, such as more efficient learning.

## Data Availability

The data that support the findings of this study are available from the corresponding author upon reasonable request.
